# Dimethylmercury Formation Mediated by Inorganic and Organic Reduced Sulfur Surfaces

**DOI:** 10.1038/srep27958

**Published:** 2016-06-15

**Authors:** Sofi Jonsson, Nashaat M. Mazrui, Robert P. Mason

**Affiliations:** 1 Department of Marine Sciences, University of Connecticut, 1080 Shennecossett Road, Groton, CT06340, USA; 2 Centre for Environment and Sustainability, University of Gothenburg, Box 170, SE-405 30, Gothenburg, Sweden

## Abstract

Underlying formation pathways of dimethylmercury ((CH_3_)_2_Hg) in the ocean are unknown. Early work proposed reactions of inorganic Hg (Hg^II^) with methyl cobalamin or of dissolved monomethylmercury (CH_3_Hg) with hydrogen sulfide as possible bacterial mediated or abiotic pathways. A significant fraction (up to 90%) of CH_3_Hg in natural waters is however adsorbed to reduced sulfur groups on mineral or organic surfaces. We show that binding of CH_3_Hg to such reactive sites facilitates the formation of (CH_3_)_2_Hg by degradation of the adsorbed CH_3_Hg. We demonstrate that the reaction can be mediated by different sulfide minerals, as well as by dithiols suggesting that e.g. reduced sulfur groups on mineral particles or on protein surfaces could mediate the reaction. The observed fraction of CH_3_Hg methylated on sulfide mineral surfaces exceeded previously observed methylation rates of CH_3_Hg to (CH_3_)_2_Hg in seawaters and we suggest the pathway demonstrated here could account for much of the (CH_3_)_2_Hg found in the ocean.

Dimethylmercury is a volatile and highly toxic form of mercury (Hg)^1^. It appears to be ubiquitous in marine waters and has been found in deep hypoxic oceanic water, coastal sediments and upwelling waters and in the mixed layer of the Arctic ocean^2^,^3^,^4^,^5^,^6^. Reported concentrations of (CH_3_)_2_Hg in marine waters range from 0.01–0.4 pM and (CH_3_)_2_Hg has been found to constitute a significant fraction (up to 80%) of the methylated Hg pool (CH_3_Hg + (CH_3_)_2_Hg)^1^,^6^. The role of (CH_3_)_2_Hg in the biogeochemical cycle of mercury, and its bioaccumulative potential, is not well known^6^,^7^,^8^. However, for oceanic systems, and for the marine boundary layer, it has been suggested that degradation of (CH_3_)_2_Hg is an important source of CH_3_Hg^5^,^9^,^10^.

Monomethylmercury (CH_3_Hg^II^X^−I^ where X is Cl^−1^, OH^−1^, R-S^−1^ etc., here referred to as CH_3_Hg) is known to bioaccumulate in aquatic food webs to concentrations of concern for human and wildlife health^1^. Understanding the methylation processes of Hg has thus been a key objective for comprehending the factors influencing its biogeochemical cycle. Formation of CH_3_Hg and (CH_3_)_2_Hg by aquatic organisms was first observed by Jensen and Jernelov in 1969^11^. A large number of bacterial strains have since been tested for their ability to methylate Hg, primarily focusing on CH_3_Hg formation. A corrinoid type protein and a 2[4Fe-4S] ferredoxin protein encoded by the HgcA and HgcB gene, respectively, was recently identified as essential for CH_3_Hg production by anaerobic bacteria^12^. The number of bacterial strains tested for their ability to methylate Hg to (CH_3_)_2_Hg is however limited and the main process remains to be identified^13^,^14^.

In culture studies with *Desulfovibrio desulfuricans,* Baldi and his coworkers, observed production of (CH_3_)_2_Hg in paralell with a white precipitate following high additions of CH_3_Hg*(aq)*^14^. This white precipitate was identified as bismethylmercury sulfide, (CH_3_Hg)_2_S*(s)*. Previous work had shown (CH_3_Hg)_2_S*(s)* formation from the reaction between CH_3_Hg(*aq*) and H_2_S, and with time, its degradation to metacinnabar (β-HgS*(s)*) and (CH_3_)_2_Hg^15^. Baldi and his coworkers thus suggested the production of (CH_3_)_2_Hg by bacteria as an effect of sulfidogenic growth. Currently, the known pathways of (CH_3_)_2_Hg formation relevant to field conditions include reaction of CH_3_Hg*(aq)* with H_2_S^15^ or selenoaminoacids^16^ and methylation with methylcobalamin^17^. Computational calculations suggest a possible formation pathway from CH_3_Hg complexed to L-cysteine, however experimental data is lacking^18^. With a up to 90% of the CH_3_Hg in marine waters naturally occurring adsorbed to reduced sulfur groups on minerals or bound to thiols on organic matter, surface mediated processes are of interest. We therefore hypothesized that (CH_3_)_2_Hg could be formed from CH_3_Hg adsorbed to inorganic and organic reduced sulfur surfaces.

## Result and Discussion

To test if (CH_3_)_2_Hg could be formed from CH_3_Hg on reduced sulfur surfaces, we initially adsorbed CH_3_Hg to disordered Mackinawite (FeS_m_*(s)*) in degassed purified water under low oxygen atmosphere and quantified the amount of (CH_3_)_2_Hg formed. During the 1 h long experiment, we detected 0.37 ± 0.08 (0–20 min), 0.21 ± 0.07 (20–40 min) and 0.16 ± 0.07 (40–60 min) pmol of (CH_3_)_2_Hg formed from 2.3 nmol of CH_3_Hg (Supplementary Table S1). Control experiments with water and filtered FeS_m_*(s)* slurry (0.02 μm) did not produce detectable levels of (CH_3_)_2_Hg supporting its formation from CH_3_Hg adsorbed onto FeS_m_*(s)* particles.

**Figure 1:**
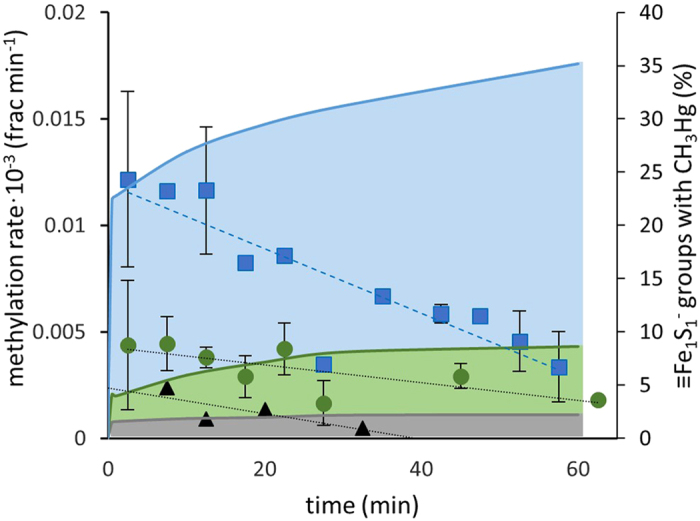
Methylation of CH_3_Hg at different CH_3_Hg:FeS_m_*(s)* ratios. Methylation rate of CH_3_Hg (fraction min^−1^, scatter plot, left hand axis) and percent of ≡Fe_1_S_1_^−^ groups on the FeS_m_*(s)* surface with CH_3_Hg adsorbed (background area graph, right hand axis) at CH_3_Hg:FeS_m_*(s)* ratios (nmol μmol^−1^) of 3.9 (blue squares, upper blue area), 1.0 (green circles, middle green area) and 0.25 (black triangles, lower gray area). Methylation rates at the three CH_3_Hg:FeS_m_*(s)* ratios tested were significantly different (p < 0.05, Analysis of Covariance).

**Figure 2:**
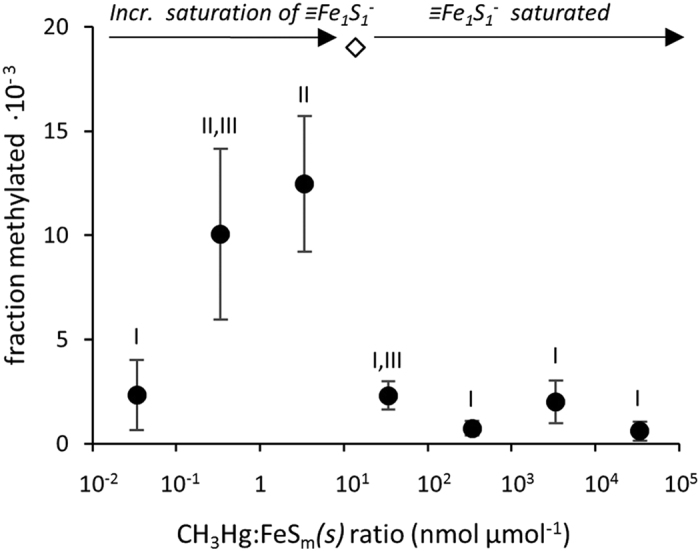
Methylation of CH_3_Hg at different CH_3_Hg:FeS_m_*(s)* ratios. Fraction of CH_3_Hg methylated at CH_3_Hg:FeS_m_*(s)* ratios (nmol μmol^−1^) of 3.4·10^−2^ to 3.4·10^4^ and the theoretical saturation point of ≡Fe_1_S_1_^−^ groups on the FeS_m_*(s)* surface (diamond). Roman numbers indicate significant differences (p < 0.05).

In the present experiment, CH_3_Hg was the only methyl containing compound and therefore acted as both the methyl donor and acceptor. The reaction could therefore involve either two CH_3_Hg molecules adsorbed on neighboring sulfide groups or one molecule adsorbed reacting with a molecule in solution. To test this, we measured the formation of (CH_3_)_2_Hg at CH_3_Hg:FeS_m_*(s)* ratios (nmol·μmol^−1^) of 6.1, 1.8 and 0.38 by varying the concentration of CH_3_Hg. FeS_m_*(s)* has been described as having a surface dominated by equal moles of mono and tri coordinated sulfide groups with the mono coordinated sulfide (≡Fe_1_S_1_^−^) having stronger anionic properties^19^. We therefore assume these are the primary sites of CH_3_Hg adsorption and calculated the fraction of ≡Fe_1_S_1_^−^ groups occupied by CH_3_Hg in the experiment. We observed a greater fraction of CH_3_Hg methylated at higher CH_3_Hg:FeS_m_*(s)* ratios; i.e. where a higher percent of ≡Fe_1_S_1_^−^ sites are saturated (Fig. 1). The fraction of CH_3_Hg in solution did not differ between the tests (Supplementary Fig. S1). This suggests the fraction methylated to be dependent on the number of sites occupied rather than concentration of CH_3_Hg remaining in solution. Additional experiments covering a wider range of CH_3_Hg:FeS_m_*(s)* ratios (3.4·10^−2^ to 3.4·10^4^ nmol·μmol^−1^), obtained by varying the concentration of FeS_m_*(s)*), demonstrated that the fraction of CH_3_Hg that was methylated increased as more ≡Fe_1_S_1_^−^ sites were occupied, and then decreased after the number of ≡Fe_1_S_1_^−^ groups were saturated with CH_3_Hg, and the fraction of CH_3_Hg bound decreased (Fig. 2). Both our experiments thus support a reaction mechanism involving two CH_3_Hg molecules adsorbed on neighboring sulfide groups rather than a reaction involving one CH_3_Hg molecule adsorbed on the surface and one molecule in solution.

FeS_m_*(s)* is the first mineral formed from environmental precipitation of S^2−^*(aq)* and Fe^2+^ *(aq);* e.g. in sediment pore water and inside bacterial cells, and is the precursor to more stable FeS forms; e.g, greigite (Fe_3_S_4_*(s)*) and pyrite (FeS_2_*(s)*)^20^. Experiments with other, more stable, sulfide minerals (CdS*(s)* and HgS*(s)*), showed similar fractions of CH_3_Hg conversion to (CH_3_)_2_Hg suggesting that the internal stability of the mineral is of minor importance (Supplementary Table S2). In a similar manner, the aging of FeS_m_*(s)* did not affect the fraction methylated (Supplementary Table S3).

**Figure 3:**
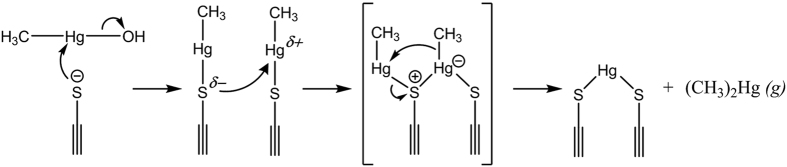
Proposed reaction mechanism. The proposed S_N_2-type reaction mechanism for the formation of (CH_3_)_2_Hg from CH_3_Hg mediated by inorganic or organic surfaces with neighboring reduced sulfur groups.

Based on the above discussed results, we propose a S_N_2-type reaction for the formation of (CH_3_)_2_Hg from CH_3_Hg adsorbed onto sulfide mineral surfaces (Fig. 3). After adsorption of CH_3_Hg onto the surface, the reaction is initiated by a nucleophilic attack of one of the CH_3_Hg holding sulfur atoms on a Hg atom of a CH_3_Hg molecule adsorbed on a neighboring sulfide site. The intermediate formed is then rearranged resulting in, as final products, one (CH_3_)_2_Hg molecule and incorporation (co-precipitation) of the other Hg atom, becoming bound to two sulfur atoms at the surface of the sulfide mineral. For the reaction of CH_3_Hg with FeS_m_*(s),* previous spectroscopic studies of Hg^2+^ adsorbed to FeS_m_*(s)* suggest that the Hg atom could either remain on the surface of the mineral or be precipitated as metacinnabar, β-HgS*(s)* (and Fe^2+^ be released into the solution)^21^,^22^. Which of these two end products would dominate in our experiment remains unclear as the final presumed Hg^2+^ :FeS_m_*(s)* is lower than in the previous studies, and furthermore, a significant fraction of added Hg is likely still remaining as CH_3_Hg adsorbed onto the FeS_m_*(s)*. Calculations of the equilibrium constant and the ΔG for the overall reaction of CH_3_Hg and FeS_m_*(s)* with (CH_3_)_2_Hg and HgS*(s)* as end-products supports that the reaction is thermodynamically favorable (see supplementary discussion).

**Figure 4:**
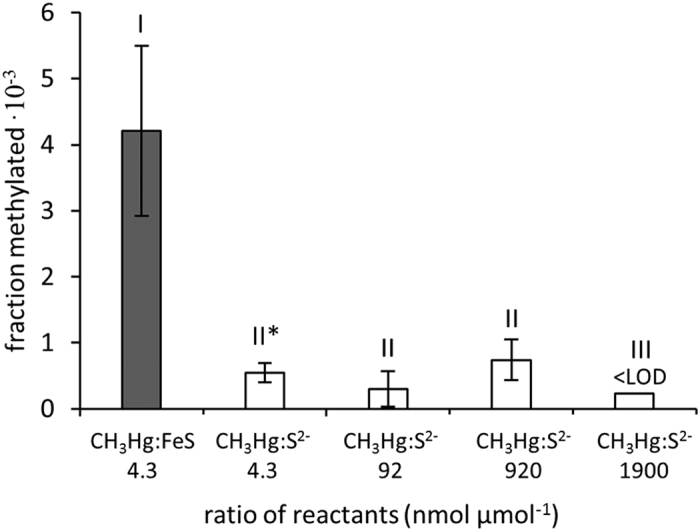
Methylation of CH_3_Hg with S^2−^*(aq)*. Fraction of CH_3_Hg methylated on FeS_m_*(s)* (±SD, n = 3) at a CH_3_Hg:FeS_m_*(s)* ratio of 4.3 (nmol μmol^−1^) or with dissolved sulfide at CH_3_Hg:S^2−^ ratios of 4.3 to 1900 (nmol μmol^−1^). LOD = Limit of detection. Roman numbers indicate significant differences (p < 0.05). *One outlier removed (n = 2).

We also tested the previously demonstrated methylation pathway involving CH_3_Hg*(aq)* and dissolved sulfide^15^ and compared it to the reaction mediated by FeS_m_*(s)*. The ratio CH_3_Hg:S^2−^ was varied from the optimum molar ratio of 2 (2 CH_3_Hg*(aq)* + S^2−^*(aq)* → HgS*(s)* + (CH_3_)_2_Hg*(g)*) to that matching the CH_3_Hg:FeS_m_*(s)* experiments (4.3 nmol μmol^−1^). The fraction CH_3_Hg methylated with S^2−^*(aq)* was 6–40 times lower than the fraction methylated on FeS_m_*(s)* (Fig. 4). The geometry of the (CH_3_Hg)_2_S molecule should theoretically limit the transfer of the methyl group between Hg atoms bound to the same S (given the linearity of the S-Hg-C bond). We found the activation energy, E_a_, for the formation of (CH_3_)_2_Hg from the reaction of CH_3_Hg with S^2−^*(aq)* and FeS_m_*(s)* to be 41 ± 6.8 and 91 ± 4.6 kJ mol^−1^, respectively (Supplementary Fig. S2). This suggests that the reaction with dissolved sulfide is slower due to a limited number of CH_3_Hg molecules close enough for transfer of the methyl group to occur. The previously proposed reaction mechanism for the observed formation of (CH_3_)_2_Hg in pure cultures of sulfate reducing bacteria, and in sediment amended with CH_3_Hg*(aq)* and purged with H_2_S*(g)*, involves (CH_3_Hg)_2_S*(s)* as an intermediate^14^,^15^. Given that surfaces with reduced sulfide would also be present in such experimental systems, we suggest that even though (CH_3_Hg)_2_S*(s)* has been observed when reacting CH_3_Hg*(aq)* with H_2_S in water and when CH_3_Hg was added to a subsample of the cell cultures, formation of (CH_3_)_2_Hg by the mechanism proposed here is also a possible explanation for the (CH_3_)_2_Hg produced in those previous experiments.

Experiments conducted from pH 6 at which most ≡Fe_1_S_1_^−^ groups would be protonated, to pH 8 where they would be deprotonated^19^, showed no difference in (CH_3_)_2_Hg formation (Supplementary Fig. S3). Further, experiments conducted at ionic strengths of 0.017 and 0.20 M (NaCl) demonstrated that ionic strength did not impact the methylation. Adsorption studies of inorganic Hg onto FeS_m_*(s)* at different pH levels have shown no significant difference in the amount of inorganic Hg adsorbed even though small differences in the dissolved fraction were observed^23^. Our results showing that (CH_3_)_2_Hg formation rate is independent of pH and ionic strength are consistent with the high binding capacity of FeS_m_*(s)* for Hg compounds in both acidic and basic conditions and the fact that the reaction between CH_3_Hg and FeS_m_*(s)* is a surface mediated process.

**Figure 5:**
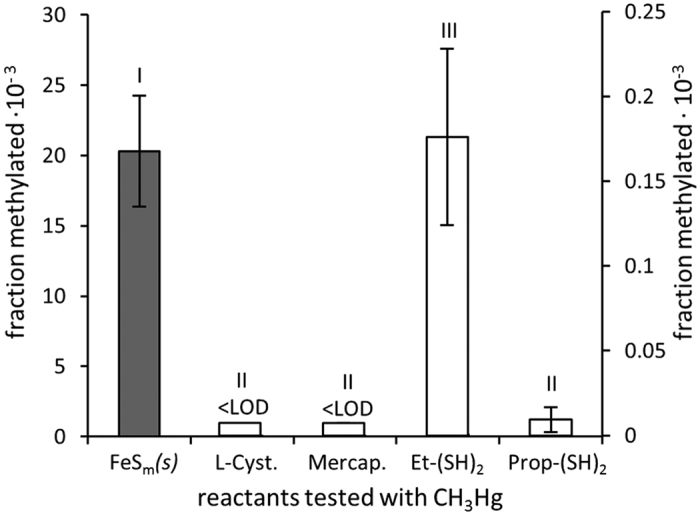
Methylation of CH_3_Hg complexed with organic thiols. Fraction of CH_3_Hg methylated (±SD, n = 3) on FeS_m_*(s)* (CH_3_Hg:FeS_m_*(s)* ratio of 3.4 nmol μmol^−1^, left hand axis) or with L-Cysteine (L-Cyst.), 3-mercaptopropionic acid (Mercap.), 1,2-ethanedithiol (Et-(SH)_2_) or 1,3-propanedithiol (Prop-(SH)_2_) (CH_3_Hg:thiol ratio of 1000 and 2000 nmol μmol^−1^ for mono- and dithiols respectively giving a CH_3_Hg:R-SH ratio of 1 for both mono and di-thiols, right hand axis), right hand axis). LOD = Limit of detection. Roman numbers indicate significant differences (p < 0.05).

Reactive sites containing multiple thiol groups located on the surface of proteins are known to be important adsorption sites for heavy metals, including Hg^24^. To test if (CH_3_)_2_Hg could also be formed from CH_3_Hg adsorbed onto neighboring thiol groups, we reacted CH_3_Hg with two dithiol compounds (1,2-ethanedithiol and 1,3-propanedithiol) and two monothiol compounds (L-cysteine and 3-mercaptopropionic acid) at CH_3_Hg:R-SH ratios of 1:1. We detected methylation of CH_3_Hg using the dithiols but not with the monothiols (i.e. fraction methylated <7.2·10^−6^) (Fig. 5). The higher methylation observed using 1,2-ethanedithiol compared to 1,3-propanedithiol could be due to a longer distance between the thiols of the latter. The fraction of CH_3_Hg being methylated was two orders of magnitude lower when the reaction was mediated by 1,2-ethanedithiol compared to FeS_m_*(s)*. The sulfidic mineral surfaces will have a higher density of electrons in comparison to alkane dithiols, which should be favorable for the proposed reaction (Fig. 3). We used simple organic dithiols here as analogs for protein sites with multiple thiol groups as previous work have shown Hg^2+^ to complex proteins and natural organic matter via thiol groups as a bicoordinated complex (RS-Hg-SR)^24^,^25^. The reactivity and symmetry (which is likely more flexible in proteins) are likely different between alkane dithiols and active sites on proteins. Nonetheless, our results show the potential for the formation of (CH_3_)_2_Hg from CH_3_Hg adsorbed on neighboring protein thiol groups. We speculate that the higher methylation rate mediated by sulfide minerals suggests that this reaction could be more favorable on iron sulfur clusters (e.g. Fe_2_S_2_, Fe_3_S_4_, Fe_4_S_4_) present in certain proteins^26^,^27^ compared to protein thiols. We examined the potential for the reaction to occur in artificial sea water in presence of diatom algae cells (*Thalassiosira weissflogii*) by comparing the formation of (CH_3_)_2_Hg in pure sea water or in sea water with the presence of whole cells, cellular membrane material and organelles (i.e. nuclei and mitochondria settled at the g forces used here), or the remaining cytoplasm (Supplementary Fig. S4). In all cases, while (CH_3_)_2_Hg was formed, the rate of formation was lower (8.6, 1.4 and 2.5 times in the presence of whole cells, cellular membrane material and organelles, respectively) than for FeS_m_*(s)* in seawater without organic matter present. The lower methylation in presence of plankton organic matter may be the result of an increase in the competition of CH_3_Hg binding to sites less reactive for the methylation process. The results however demonstrate the potential for the above outlined mechanism to occur on FeS-clusters within cells after assimilation of CH_3_Hg from marine waters.

Our study is the first to demonstrate the formation of (CH_3_)_2_Hg from CH_3_Hg adsorbed onto sulfide mineral surfaces or organic dithiols (CH_3_Hg methylation rates up to 0.012 ± 0.004 × 10^−3^ detected, Fig. 1). In the ocean, the highest concentrations of (CH_3_)_2_Hg are typically found in low oxygen environments where active degradation of organic matter is occurring, or in regions of concentrated biological material, as well as in the deep ocean^1^,^2^. The relatively high degradation rate of (CH_3_)_2_Hg observed in marine waters suggests it must be continually produced in the water column, sediments or in association with hydrothermal systems^7^. The formation of (CH_3_)_2_Hg has mainly been hypothesized to be bacterially mediated, however direct experimental support for this assertion is missing^28^. Further, the *in vivo* mechanism by which the (CH_3_)_2_Hg could be produced inside the bacterial cells has not been identified^14^. We propose that the reaction pathway discussed above may be important abiotic as well as biotic pathways for formation of (CH_3_)_2_Hg in the oceanic system. In addition to methylation of CH_3_Hg in the presence of biological material via pathway involving the binding of CH_3_Hg to thiols and the proposed methyl transfer reactions outlined above, there is also the potential for these reactions to occur in the presence of metal-sulfide particles within marine aggregates, or in the sulfide particles that are associated with hydrothermal vent plumes. The presence of reduced sulfur in the upper ocean has been shown in numerous studies^29^,^30^. There is also evidence for reducing conditions within sinking marine aggregates, and the presence of CdS*(s)* in low oxygen sub-thermocline ocean waters^31^,^32^. Finally, there is substantial evidence for metal sulfide and pyrite particulates emanating from hydrothermal vents^33^. Although the concentrations of CH_3_Hg in our experiments exceed the concentrations found in marine waters, our ratio of CH_3_Hg to sulfide mineral surface area are similar to the ratio expected on particles or inside planktonic cells present in the ocean. For example, for the low oxygen waters in the North Atlantic, where observed concentration of particulate Cd has been assumed to mainly be composed of CdS*(s)*, calculated particulate CH_3_Hg:Cd is about 10^−3^ (molar basis)^34^,^35^. Furthermore, inside planktonic cells the molar CH_3_Hg:Fe ratio of 10^−3^ to 10^−4^ is typically found but the expected CH_3_Hg:FeS is lower given that not all intracelleular Fe would occur as FeS-clusters^8^,^36^,^37^. Reported rates of (CH_3_)_2_Hg formation in marine water are scant. Lehnherr *et al.* reported potential CH_3_Hg methylation rates, producing (CH_3_)_2_Hg, of up to 1.6·10^−3^ d^−1^ for Arctic waters^28^. The fraction of CH_3_Hg converted to (CH_3_)_2_Hg in our experiments (up to 20·10^−3^ in purified water and up to 15·10^−3^ in artificial sea water, Fig. 5 and Supplementary Fig. S4) for experiments carried out within 24 h are an order of magnitude higher than the methylation rates observed by Lehnherr *et al.* We propose that the reactions outlined above could produce a significant portion of the (CH_3_)_2_Hg within the upper ocean water column, primarily in association with organic matter recycling. In the deep ocean, the elevated concentrations of total Hg, CH_3_Hg, as well as dissolved and colloidal Fe, found during the Geotraces GA03 cruise in the vicinity of the mid-Atlantic Ridge^34^,^38^, compared to other deep ocean waters, suggest that hydrothermal vent plumes are environments where (CH_3_)_2_Hg could be formed by reactions mediated by FeS surfaces.

## Material and Methods

The preparation of sulfide minerals and all experiments were conducted under a N_2_*(g)* or Ar*(g)* atmosphere and using degassed (N_2_*(g)* or Ar*(g)* purged) purified water (Ω < 18.2). Disordered Mackinawite (FeS_m_*(s)*) was prepared by adding 100 ml of 0.6 M Na_2_S to 100 ml of 0.6 M Morh’s salt ((NH_4_)_2_Fe(II)(SO_4_)_2_∙6H_2_O)^39^. The precipitated crystals of FeS_m_*(s)* were aged for 0 h, 1 day and 7 days, then collected by centrifugation (5 min, 2.6 kG) and washed three times with purified water. Since the aging of FeS_m_*(s)* has previously been shown to significantly stop at −80 °C^39^, the final product was re-suspended in water, subsampled into smaller vials and stored in a N_2_ atmosphere at −80 °C until use. For experiments where the activity of FeS_m_*(s)* was compared to that of CdS*(s)* and HgS*(s)* (Supplementary Table S2), FeS_m_*(s)* was prepared as described above (25 ml of 0.6 M Na_2_S and 0.6 M of Morh’s salt), and at the same time, CdS*(s)* and HgS*(s)* were synthesized by adding 25 ml or 15 ml of 0.6 M Na_2_S to 25 ml of 0.6 M Cd(NO_3_)_2_·4 H_2_O or 15 ml 0.6 M HgCl_2_ (prepared by dissolving HgCl_2_*(s)* using 700 μL HCl following dilution in purified water), respectively. For CdS*(s)* precipitated with excess of Cd, this was prepared by adding 12.5 ml of 0.6 M Na_2_S and 12.5 ml degassed MQ water to 25 ml 0.6 M Cd(NO_3_)_2_·4 H_2_O. The precipitated crystals were collected by centrifugation (5 min, 2.6 kG), and washed four times until excess acid in the HgS*(s)* slurry was removed (pH ~7). A subsample of each was freeze-dried to calculate the concentration (weight to weight) of stock slurries, and characterized using X-ray Diffraction Cristallography (XRD) and Brunauer–Emmett–Teller (BET) (Supplementary Table S4, Fig. S5 and discussion).

Formation of (CH_3_)_2_Hg was tested by adding CH_3_Hg*(aq)* to FeS_m_*(s)*, CdS*(s)*, HgS*(s)*, S^2−^*(aq)*, L-Cysteine, 3-mercaptopropionic acid, 1,2-ethanedithiol or 1,3-propanedithiol in acid cleaned glass vials (total volume of 42 cm^3^). The amount of thiol ligand, CH_3_Hg and final volume of solution used is summarized in Supplementary Table S5. Each experimental set was done in triplicate and the CH_3_Hg*(aq)* standard was prepared from a 1000 ppm CH_3_Hg*(aq)* stock solution (pH 1, Alfa Aesar) and pH was adjusted to ~6–8 using 2–8 M KOH*(aq)*. The produced (CH_3_)_2_Hg*(g)* was collected onto Carbotrap^TM^ (Supelco) solid absorbent either by purging the headspace of the vial with 200 ml/min of Argon (Ar) while gently stirring the solution with a magnetic stirring bar (results presented as formation rates, i.e. n(CH_3_)_2_Hg(g) (pmol) · nCH_3_Hg added (pmol)^−1^ · time (min)^−1^), or by sampling 0.1–5 ml of the headspace from a closed vial through the septa using a syringe. For the latter, the total concentration of (CH_3_)_2_Hg was calculated based on the relative volumes of water and gas, the sampled volume of gas and the dimensionless Henry solubility constant (H^cc^; concentration in the aqueous phase · concentration in the gas phase^−1^) for (CH_3_)_2_Hg^40^, and results are presented as fraction of CH_3_Hg methylated (i.e. n(CH_3_)_2_Hg(g) (pmol) · nCH_3_Hg added (pmol)^−1^). In the initial experiment, the FeS_m_*(s)* slurry was filtered through a 0.02 um PTFE syringe filter and the control experiment done by adding 2.3 nmol of CH_3_Hg to 1 ml of the filtrate. The percent of ≡ Fe_1_S_1_^−^ with CH_3_Hg adsorbed was calculated from the concentration of CH_3_Hg*(aq)* immobilized in a separate adsorption experiment (Supplementary Fig. S1), the specific surface area of FeS_m_*(s)* (Supplementary Table 3) and assuming two ≡ Fe_1_S_1_^−^ -groups nm^−2 ^19. The activation energy, E_a_ (kJ/mol), for the formation of (CH_3_)_2_Hg was determined assuming a pseudo first order reaction and using the Arrhenius Equation (ln*k* = ln*Ae* – E_a_/RT; rate constant (*k*), frequency factor (*Ae*), activation energy (E_a_), gas constant (R), temperature (T; in kelvin) from experiments conducted at 0, 18, 40 and 60 °C (n = 3, details are provided in Supplementary Table S5). The activation energy (including standard deviation) was calculated from the slope of ln k vs. 1/T (slope = −E_a_/R). For CH_3_Hg on FeS_m_*(s)*, no (CH_3_)_2_Hg*(g)* was detected in samples incubated at 0 °C hence the production of (CH_3_)_2_Hg during the cooling process was neglected. For the reaction with S^2−^ (where a higher concentration of CH_3_Hg was used), the (CH_3_)_2_Hg formed was similar at 0 and 18 °C. The E_a_ was thus calculated only using the results obtained at 40 and 60 °C.

When the reaction vessels were purged, sampled gas was first dried on a soda lime trap placed in line with the Carbotrap^TM^ column, and when the headspace was sampled, the gas was injected directly on the Carbotrap^TM^ column via an injection valve. Collected (CH_3_)_2_Hg*(g)* was then thermally desorbed and separated by isothermal gas chromatography before being pyrolytically decomposed to Hg^0^ and detected using CVAFS (Tekran, model 2500). External calibration was done using known amounts of synthesized (CH_3_)_2_^200^Hg*(aq)* standard purged onto Carbotrap^TM^ columns. The (CH_3_)_2_^200^Hg was manufactured in house from ^200^HgCl_2_ and 3 M methyl magnesium chloride in tetrahydrofuran (Supplementary Method). WARNING, Extreme caution is needed when synthesizing (CH_3_)_2_Hg as it is a volatile and extremely toxic compound! Even small amounts absorbed through the skin have proven fatal! Due to variations in the concentration of (CH_3_)_2_Hg in the diluted aqueous standard prepared from synthesized stock solution, standards were prepared daily and the concentration was determined by collecting purgeable Hg from the standard on to a gold trap and using a second calibration of 10–200 ul of Hg^0^*(g)* at a known temperature (also purged onto gold traps). Detection limits were calculated from the amount of (CH_3_)_2_Hg detected from experimental replicates utilizing equimolar concentrations of CH_3_Hg*(aq)* (n = 3, mean + 2 SD).

Adsorption of CH_3_Hg*(aq)* onto FeS_m_*(s)* was tested by incubating 0.70 (n = 1), 2.8 (n = 1) and 11 (n = 1) nmol of CH_3_Hg with 2.8 umol FeS_m_*(s)* in 0.6 ml of DI in a disposable syringe. The samples were left for up to 60 minutes and the dissolved fraction was then collected using a 0.02 um syringe filter. In a second experiment, 0.34 nmol CH_3_Hg was added to 50 μmol of FeS_m_*(s)* in 10 ml degassed DI. The samples were filtered after an equilibration time of 10 min, 60 min or 24 h using 0.05 um membrane filters. The amount of CH_3_Hg remaining in solution was quantified using EPA method 1630 with an automated analyzer (Tekran 2700). Statistical analysis was conducted using IBM SPSS statistics. All data (fraction methylated or methylation rates) were log transformed before analysis of variance followed by Tukey’s post-hoc test. For non-normally distributed log transformed data, median test following a pairwise t-test approach was performed.

## Additional Information

**How to cite this article**: Jonsson, S. *et al.* Dimethylmercury Formation Mediated by Inorganic and Organic Reduced Sulfur Surfaces. *Sci. Rep.* **6**, 27958; doi: 10.1038/srep27958 (2016).

## Supplementary Material

Supplementary Information
