# Supplementary material for: Dimethylmercury Formation Mediated by Inorganic and Organic Reduced Sulfur Surfaces
Source: Sci Rep. 2016 Jun 15;6:27958. doi: 10.1038/srep27958 (PMC4908375; doi:10.1038/srep27958)
Supplement: Supplementary Information [file srep27958-s1.pdf]

Supplementary Information for

Dimethylmercury Formation Mediated by Inorganic and Organic Reduced Sulfur Surfaces

Sofi Jonsson[1,2]*, Nashaat M. Mazrui[1)], Robert P. Mason[1)]

[1)] Department of Marine Sciences, University of Connecticut, 1080 Shennecossett Road, Groton, CT06340, USA

[2)] Center of Environmental and Sustainability, University of Gothenburg, Box 170, SE-405 30, Gothenburg, Sweden

***Contact information**

Department of Marine Sciences,

University of Connecticut, 1080 Shennecossett Road

Groton, CT06340, USA,

work phone: +1 860 4059281

email: sofijon84@gmail.com

Supplementary Discussion

Adsorption of $CH_3Hg(aq)$ onto FeS_m*(s)*. In the first adsorption experiment, we monitored the adsorption of 1, 5.2 and 21 μM of CH_3Hg on 0.26 g L^{-1} FeS_m*(s)* for 1 h (Supplementary Fig S1). At all three concentrations, the adsorption was initially rapid with 40-63 % of the added CH_3Hg*(aq)* immobilized within minutes, followed by a slower and gradual increase in adsorption of the CH_3Hg. At the end of the 1 h experiment the percent CH_3Hg removed from solution was 89, 88 and 91% of added CH_3Hg, corresponding to log K_D values of 4.5, 4.4 and 4.6 L Kg^{-1}, for the test with 1, 5.2 and 21 μM of CH_3Hg respectively. The obtained log K_D values are within range of the typically reported values from aquatic systems[1]. A repeated experiment carried out for up to 24 h and using 0.05 μm membrane filters, instead of 0.02 μm syringe filters, showed a similar pattern of immobilization (Supplementary Fig S1; bar graph).

Characterization of synthesized particles. Crystal structures of the synthesized FeS_m*(s)*, CdS*(s)*, and HgS*(s)* particles was determined using X-ray diffraction (Supplementary Fig S5) and BET, respectively (Supplementary Table S3). The XRD diffractograms were consistent with the diffraction patterns of ordered tetragonal mackinawite, hexatetrahedral hawleyite and cubic metacinnabar, respectively. The FeS_m*(s)* diffractogram shows broad and less intense peaks compared to those of CdS(s) and HgS(s). This is indicative of small crystallites size and high degree of amorphousness. A high degree of disorder in the synthetic FeS_m*(s)* is in agreement with existing literature[2].

Thermodynamic favorability of the reaction. The equilibrium constant for the overall reaction of CH_3Hg with FeS_m*(s)* (reaction below) assuming HgS*(s)* as a final product was calculated from stability constants provided in Stumm and Morgan (1996)[3]. At the concentrations used in the experiments, and assuming the Fe^{2+} concentration is equivalent to that at equilibrium with the solid phase, at a pH of 7, the overall ΔG for the reaction is ~150 kJ mol^{-1} at STP. This indicates a highly favorable reaction.

$$2\ CH_3HgOH + 2H^+ + FeS_m(s) \rightarrow HgS(s) + (CH_3)_2Hg + Fe^{2+} + H_2O \qquad \log K\ 37.7$$

Supplementary Figures

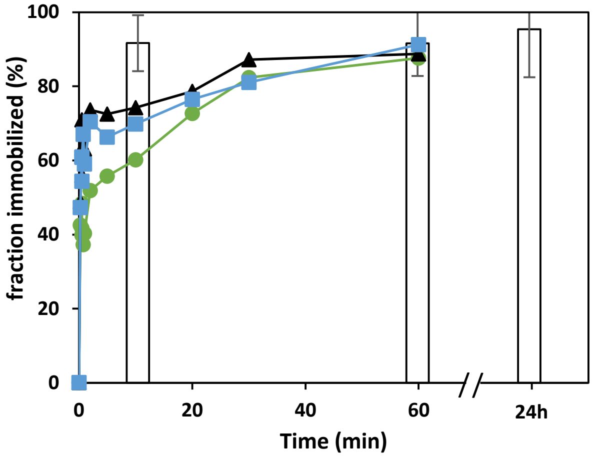

Supplementary Fig. S1. Adsorption of CH_3Hg*(aq)* on FeS_m*(s)*. Fraction CH_3Hg*(aq)* immobilized by FeS_m*(s)* as a function of time in an experiment using 0.02 µm syringe filters and CH_3Hg:FeS_m ratios (nmol μmol^{-1}) of 3.9 (blue squares), 1.0 (green circles) and 0.25 (black triangles) or using 0.05 µm membrane filters (bar graph) and a $(CH_3)_2Hg$:FeS_m ratio of 0.068 nmol μmol^{-1}.

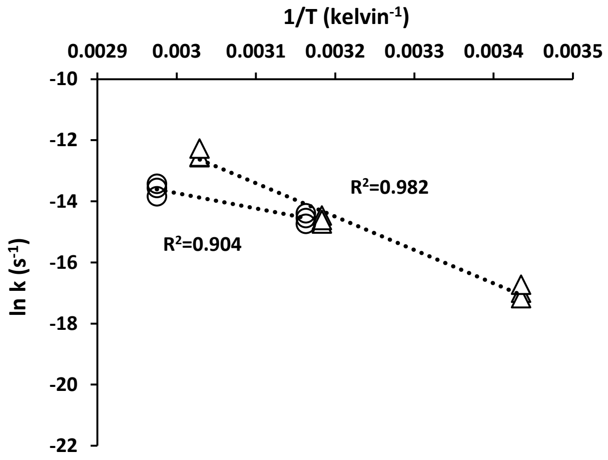

Supplementary Fig. S2. Methylation of CH_3Hg on FeS_m*(s)* at different temperatures. Effect of temperature (1/T (kelvin^{-1})) on the reaction rate constant (k, s^{-1}) for 7.5 nmol CH_3Hg added to 150 µmol FeS_m*(s)* (triangles) and 390 nmol CH_3Hg*(aq)* reacted with 380 nmol S^{2-}*(aq)* (circles). Both regression models were statistically significant (linear regression, ANOVA, $p<0.05$). Activation energy, E_a (kJ/mol), for the formation of $(CH_3)_2Hg$ was determined assuming a pseudo first order reaction and using the Arrhenius Equation ($\ln k = \ln Ae - E_a/RT$; rate constant (*k*), frequency factor (*Ae*), activation energy (Ea), gas constant (R), temperature (T; in kelvin). The activation energy (including standard deviation) was calculated from the slope of ln k vs. 1/T (slope = -Ea/R).

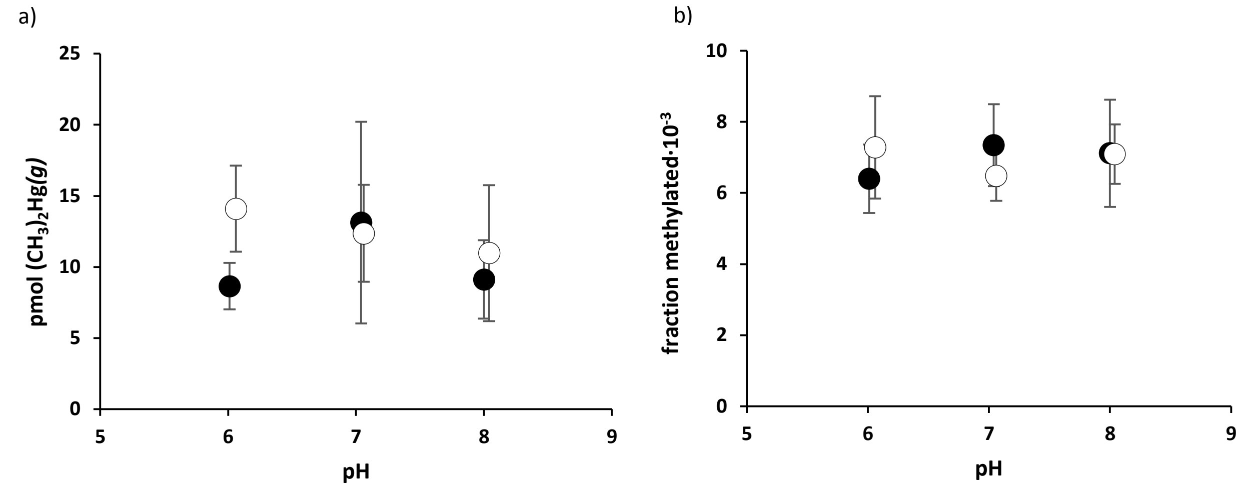

Supplementary Fig. S3. Methylation of CH_3Hg on FeS_m*(s)* at different pH and ionic strengths. Formation of $(CH_3)_2Hg$ when adsorbing CH_3Hg on FeS_m*(s)* at pH of 6, 7 or 8 and ionic strength of 0.017 (white circles) or 0.20 M (black circles) in a) short term and b) long term experiments. No significant differences (two-way ANOVA, $p>0.05$) were found for the amount of $(CH_3)_2Hg$ formed in a) or b).

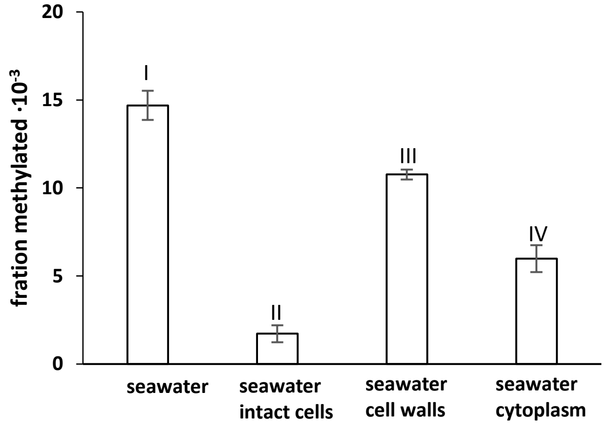

Supplementary Fig. S4. **Methylation of CH_3Hg on FeS_m*(s)* in sea water in the presence of organic material.** Fraction of CH_3Hg methylated when adsorbing CH_3Hg on FeS_m*(s)* in artificial sea water, and in artificial sea water with whole cells, cell walls (pellet obtained at 754 G for 15 min) or the cytoplasm (remaining in solution after centrifugation at 754 G, 15 min) of $1.2{\cdot}10^6$ cells of *Thalassiosira weissflogii*. Roman letters indicate significantly differences ($p<0.05$, ANOVA followed by Tukey's post-hoc test).

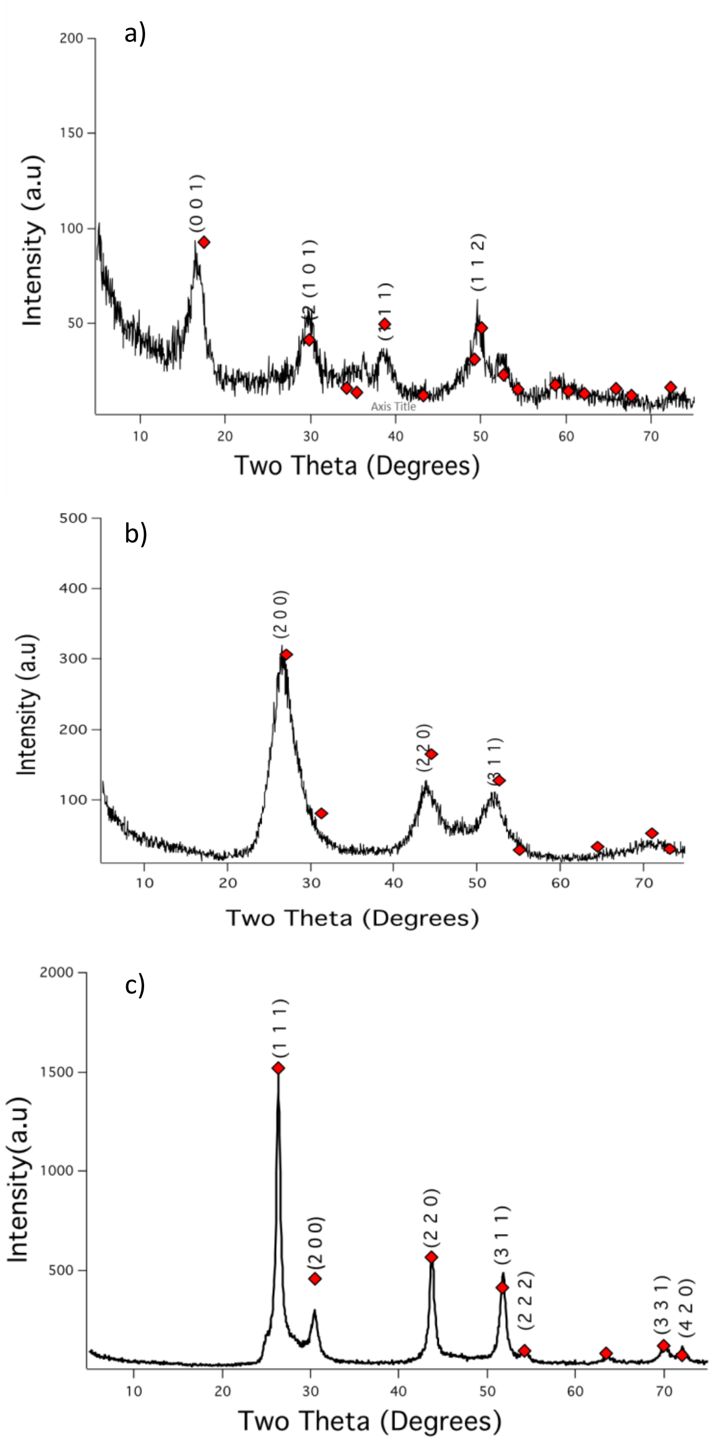

Supplementary Fig. S5. Characterization of crystal structure. X-ray diffractograms of synthesized a) FeS_m*(s)*, b) CdS*(s)* and c) HgS*(s)* with diffraction patterns (red diamonds) for ordered tetragonal mackinawite[4], hexatetrahedral Hawleyite[5] and cubic metacinnabar[4], respectively.

Supplementary Tables

Supplementary Table S1. Production of $(CH_3)_2Hg$*(g)* from CH_3Hg*(aq)* added to filtered and unfiltered FeS_m*(s)* slurries. Amount of $(CH_3)_2Hg$*(g)* (pmol) formed from 2.3 nmol of CH_3Hg*(aq)* added to 0.2 um filtrated and unfiltered FeS_m*(s)* slurries. The unfiltered FeS_m*(s)* slurry contained 5.6 umol FeS_m(s) giving a CH_3Hg:FeS_m*(s)* ratio of 0.41 (nmol $umol^{-1}$).

Sample	Purging interval (min)	n$(CH_3)_2Hg$*(g)* (pmol)
Filtrated slurry	0-20	<0.038
FeS_m*(s)*	0-20	0.37 ± 0.08
	20-40	0.21 ± 0.07
	40-60	0.16 ± 0.07

Supplementary Table S2. **Methylation of CH_3Hg on FeS_m*(s)*, CdS*(s)* and HgS*(s)*.** Fraction of CH_3Hg methylated on CdS*(s)*, FeS*(s)* and HgS*(s)* at equal CH_3Hg to specific surface area ratios (9.6 nmol CH_3Hg, 970 nmol CH_3Hg m^{-2} mineral surface). No significant differences were found for the methylation on the sulfide minerals tested (ANOVA, $p>0.05$).

	Fraction methylated $\cdot 10^{-3}$	CH_3Hg:(Fe/Cd/Hg)S*(s)* ratio	
		nmol μmol^{-1}	nmol surface area $(m^2)^{-1}$
FeS_m*(s)*[4]	8.3 ± 3.6	4.6	970
CdS*(s)*[4]	2.2 ± 1.3	10	970
CdS*(s)*[5]	6.3 ± 4.3	18	970
HgS*(s)*[4]	4.0 ± 1.9	4.3	970

[1)] *Precipitated at equimolar amounts of metal and sulfide*
[2)] *Precipitated at a Cd:S ratio of 2:1*

Supplementary Table S3. **Methylation of CH_3Hg on FeS_m*(s)* of different age.** Fraction of CH_3Hg methylated when the reaction was mediated by FeS_m*(s)*, aged for 1hour, 1day or 7 days. The amounts of CH_3Hg and FeS_m*(s)* reacted was 9.6 nmol and 2.8 μmol respectively, giving a final CH_3Hg:FeS_m*(s)* ratio of 3.4 nmol μmol^{-1}. No statistical differences between the reactions with the FeS_m*(s)* of different ages ($p>0.05$) was observed. Change in the internal structure of synthetic FeS_m*(s)* has previously been demonstrated to occur within hours to days[6].

	Fraction methylated $\cdot 10^{-3}$
FeS_m*(s)* aged 1h	16 ± 4.6
FeS_m*(s)* aged 1d	17 ± 14
FeS_m*(s)* aged 7d	12 ± 3.3

Supplementary Table S4. **Surface area of FeS_m*(s)*, CdS*(s)* and HgS*(s)*.** Specific surface areas of precipitate crystals determined using BET.

	Specific surface area (m^2 g^{-1})
FeS_m*(s)*[1, 2]	34.9
FeS_m*(s)*[1]	55.3
CdS*(s)*[1]	73.9
CdS*(s)*[3]	126
HgS*(s)*[1]	19.29

[1)] *Precipitated with equimolar amounts of metal and sulfide*
[2)] *FeS_m(s) batch used for all experiments except data presented in Supplementary Table S2.*
[3)] *Precipitated with a Cd:S ratio of 2:1*

Supplementary Table S5. Summary of experimental details. Form and amount of sulfide used to mediate the methylation, the initial amount of CH_3Hg and the final total volume of the aqueous solution used.

Test[1)]	Sulfide form		CH_3Hg	CH_3Hg:sulfide form	Volume
	name	(µmol)	(nmol)	(nmol umol^{-1})	(ml)
Initial test (text)	FeS_m*(s)*	5.6	2.3	0.41	1.1
Varying CH_3Hg (fig 1)	FeS_m*(s)*	2.8	0.7, 2.8, 11	0.25, 1.0, 3.9	0.6
Varying FeS_m*(s)* (fig 2)	FeS_m*(s)*	0.0028 to 280	9.6	3400 to 0.034	0.6
FeS_m*(s)* and S^{2-}*(aq)* (fig 4)	FeS_m*(s)*	2.8	12	4.3	0.6
	S^{2-}*(aq)*	2.8, 0.13, 0.013, 0.0063	12	4.3, 92, 920, 1900	0.6
Test of thiols[2)] (fig 5)	FeS_m*(s)*	2.8	9.6	3.4	0.6
	Cysteine	0.2	200	1000	0.6
	3-mercaptopropionic acid	0.2	200	1000	0.6
	1,2-ethanedithiol	0.1	200	2000	0.6
Temperature effect[3)] (fig S2)	FeS_m*(s)*	2.8	7.5	2.7	0.6
	S^{2-}*(aq)*	0.38	390	1000	0.6
pH and ionic strengh[4)] (fig S3)	FeS_m*(s)*	2.8	8	2.9	0.6
Artificial sea water and *t.w.*[5)] (fig S4)	FeS_m*(s)*	2.8	9.6	3.4	2.5
Different sulfide minerals[6)] (table	CdS*(s)*	0.93	9.6	10	0.6
S2)	CdS*(s)*[7)]	0.54	9.6	17	0.6
	FeS_m*(s)*	2.0	9.6	4.8	0.6
	HgS*(s)*	2.2	9.6	4.4	0.6
FeS_m*(s)* aging (table S3)	FeS_m*(s)* (aged 1h, 1d, 7d)	2.8	9.6	3.4	0.6

1) figure or table where the data is presented is given in brackets.

2) tests performed in 500 uL 0.8% (v:v) acetone in degassed MQ water

3) Sealed experimental vessels were incubated for 30 min in a water bath at a temperature of 0, 18, 40 and 60 ºC (n=3). The reaction vessels were cooled on ice for 30 minutes and before $(CH_3)_2Hg$*(g)* was sampled from the headspace.

4) prepared in 0.002 M phosphate buffer at pH of 6, 7 or 8 and ionic strength of 0.2 or 0.016 M (set with NaCl)

5) Artificial sea water was prepared as described by Kester et al.[7]. Axenic cultures of the diatom *Thalassiosira weissflogii* (t.w; culture 1.26-15, National Oceanic and Atmospheric Administration, Milford, US) were grown at f/4 for trace metals and f/2 for other nutrients[8]. The culture was purged with N_2 to remove the excess oxygen ("sea water intact cells") and a subsample was then sonicated at cycles of 3 seconds ON:OFF for 2 minutes while cooling on ice. The cell wall material and organelles (i.e. nuclei and mitochondria) was then isolated from the cytoplasm ("sea water cytoplasm") by centrifugation at 754 G[9] for 15 min and resuspended in artificial sea water ("sea water cell walls"). Formation of $(CH_3)_2Hg$ from CH_3Hg and FeS_m*(s)* was tested by incubating 100 µL of a CH_3Hg*(aq)* solution with 500 µL of a FeS_m*(s)* slurry in 2 ml of artificial sea water or the "sea water intact cells", "sea water cell wall" or "sea water cytoplasm" media.

6) the amount of the minerals was adjusted to give a surface area of $9.9 \cdot 10^{-3}$ m^2

7) CdS*(s)* precipitated with excess of Cd (Cd:S ratio of 2:1)

Supplementary Methods

Synthesis of $(CH_3)_2{}^{200}Hg$*(aq)*. The $(CH_3)_2{}^{200}Hg$ standard was manufactured in house from $^{200}HgCl_2$ and 3M methyl magnesium chloride in tetrahydrofuran (Alfa Aesar)[10]. A $^{200}HgCl_2$*(aq)* solution was heated until dryness in a glass tube. The tube was then put in an ice bath, 3M methyl magnesium chloride in tetrahydrofuran was added and the reaction was left overnight at room temperature. The reaction was cooled on ice before quenching the reaction by addition of water saturated with NaCl (exothermic reaction, added dropwise). The organic layer (tetrahydrofuran containing $(CH_3)_2{}^{200}Hg$) was then extracted using a Pasteur pipette and transferred to a new tube which was stored at -4°C. Working solutions of $(CH_3)_2{}^{200}Hg$*(aq)* were prepared by dilution of the stock solution in purified MQ.

Supplementary References

1. Fitzgerald, W. F., Lamborg, C. H. & Hammerschmidt, C. R. Marine Biogeochemical Cycling of Mercury. *Chem. Rev.* **107,** 641–662 (2007).
2. Rickard, D. & Luther, G. W. *Chemistry of iron sulfides*. *Chem. Rev.* **107,** (2007).
3. Stumm, W. & Morgon, J. J. *Aquatic chemistry: Chemical equilibrium and rates in natural waters*. (Wiley, 1996).
4. NIST Standard Reference Database 84.
5. Downs, R. & Hall-Wallace, M. The American Mineralogist crystal structure database. *Am. Mineral.* **88,** 247–250 (2003).
6. Wolthers, M., Van Der Gaast, S. J. & Rickard, D. The structure of disordered mackinawite. *Am. Mineral.* **88,** 2007–2015 (2003).
7. Kester, D., Duedall, I. W., Connors, D. & Pytkowicz, R. Preparation of Artifical Seawater. *Limnol. Oceanogr.* **12,** 176–179 (1967).
8. Guillard, R. & Ryther, J. Studies of marine planktonic diatoms. I. Cyclotella nana Hustedt and Detonula confervacea (cleve) Gran. *Can. J. Microbiol.* **8,** 229–239 (1962).
9. Fisher, N. S., Burns, K. A., Cherry, R. D. & Heyraud, M. Acculumation and cellular distribution of ^{241}Am, ^{210}Po, and ^{210}Pb in to marine algae. *Mar. Ecol.* **11,** 233–237 (1983).
10. Snell, J. P., Stewart, I. I., Sturgeon, R. E. & Frech, W. Species specific isotope dilution calibration for determination of mercury species by gas chromatography coupled to inductively coupled plasma- or furnace atomisation plasma ionisation-mass spectrometry. *J. Anal. At. Spectrom.* **15,** 1540–1545 (2000).
